# Exploring the effects of achievement emotions on online learning outcomes: A systematic review

**DOI:** 10.3389/fpsyg.2022.977931

**Published:** 2022-09-09

**Authors:** Rong Wu, Zhonggen Yu

**Affiliations:** Faculty of Foreign Studies, Beijing Language and Culture University, Beijing, China

**Keywords:** positive achievement emotions, negative achievement emotions, online learning outcomes, interventions, online learners

## Abstract

Recently, achievement emotions have attracted much scholarly attention since these emotions could play a pivotal role in online learning outcomes. Despite the importance of achievement emotions in online education, very few studies have been committed to a systematic review of their effects on online learning outcomes. This study aimed to systematically review studies examining the effects of achievement emotions on online learning outcomes in terms of motivation, performance, satisfaction, engagement, and achievement. According to the selection process of Preferred Reporting Items for Systematic Review and Meta-analysis (PRISMA) principles, a total of 23 publications were included in this review. It was concluded that positive achievement emotions, such as enjoyment, pride, and relaxation, could generally exert a positive effect on online learning motivation, performance, engagement, satisfaction, and achievement. It should be noted that excessive positive emotions might be detrimental to online learning outcomes. On the other hand, it has been difficult to determine the effects of negative achievement emotions on online learning outcomes because of disagreement on the effects of negative achievement emotions. In order to improve online learners' learning outcomes, instructors should implement interventions that help online learners control and regulate their achievement emotions. Teaching interventions, technological interventions, and treatment interventions could benefit online learners emotionally and academically. Future studies could examine the moderating roles of contextual factors and individual variables in the effects of achievement emotions on online learning outcomes.

## Introduction

### The importance of achievement emotions

Achievement emotions could not only be the consequences of achievement activities and outcomes but also play an important role in subsequent learning (Pekrun et al., 2017; Pan et al., 2022). The effects of achievement emotions on learning have attracted considerable attention, both scholarly and popular (Camacho-Morles et al., 2021). Evidence has suggested that achievement emotions could exert an important influence on learners' problem-solving ability (Lee and Chei, 2020), learning persistence (Tang et al., 2021), engagement (Luo and Luo, 2022), motivation (Feraco et al., 2022), satisfaction (Wu et al., 2021b), and achievement (Putwain et al., 2022).

### Achievement emotions in online learning

Online learners' achievement emotions have caught attention since online learning has become a new norm in recent years (Wang et al., 2022). Compared to traditional face-to-face learning, online learning has brought learners new opportunities and challenges, such as flexibility, isolation, and technical problems (Kim et al., 2014; Yu, 2021). Facing opportunities and challenges specific to online learning, learners may experience achievement emotions more frequently than those in traditional face-to-face learning (Moneta and Kekkonen-Moneta, 2007; D'Mello and Graesser, 2012; Lee and Chei, 2020). Students were more likely to experience a high level of frustration in online learning due to technical problems, compared to face-to-face learning (Hamilton et al., 2021). Nevertheless, the extant literature has mainly focused on achievement emotions in traditional face-to-face learning (Raccanello et al., 2020). Research on achievement emotions in online learning has been relatively scarce (Yang et al., 2021).

### Roles of achievement emotions in online learning outcomes

Recent research has suggested that achievement emotions could play a crucial role in online learning outcomes (Lee and Chei, 2020). Generally, positive emotions could be conducive to online learning outcomes, while negative emotions could be detrimental to online learning outcomes (Pan et al., 2022). Learners experienced positive achievement emotions (e.g., joy and relief) when gaining a great deal of the flexibility of online learning (Zembylas et al., 2008). Learners with positive achievement emotions possibly had more online learning satisfaction (Wu et al., 2021b). In contrast, many learners were less likely to attend online classes when feeling boring and disengaged due to the lack of interactive activities and emotional support in online learning (Tzafilkou et al., 2021).

However, literature has emerged that offered contradictory findings on the effects of achievement emotions (e.g., Golding and Jackson, 2021). The effects of achievement emotions on online learning outcomes may be more complex than once thought, resulting from close intertwinement with affective, cognitive, and contextual factors. (Artino and Jones, 2012; Marchand and Gutierrez, 2012). On one aspect, empirical evidence seemed to suggest the positive effects of negative emotions on online learning outcomes (e.g., Hilliard et al., 2020). On the other aspect, recent research found that positive achievement emotions had a negative influence on online learners' learning outcomes (Liu et al., 2021). Therefore, there has been little agreement on whether achievement emotions could be beneficial or detrimental to online learning outcomes.

Although recent years have witnessed an exponential growth in research on achievement emotions, one of the biggest challenges might be to summarize and synthesize the contradictory findings in this field (Camacho-Morles et al., 2021). Relatively little research has conducted a systematic review of achievement emotions, and even less on the effects of achievement emotions on online learning outcomes in terms of motivation, engagement, achievement, satisfaction, and performance. This study, aiming to synthesize available findings on the effects of achievement emotions on online learning motivation, engagement, performance, satisfaction, and achievement, is thus meaningful.

## Theoretical framework

The control-value theory (CVT) is the main theoretical framework for understanding achievement emotions (Pekrun, 2006). CVT integrates assumptions from several theories, including the expectance value theory of emotions, transactional approaches, attributional theories, and models of the effects of emotions (Pekrun et al., 2017). CVT explains how achievement emotions subsequently influence learners' motivation, performance, engagement, satisfaction, and achievement in various academic contexts (Pekrun et al., 2011). Moreover, CVT suggests that individuals' achievement emotions may vary in intensity and frequency due to gender, age, and culture (Camacho-Morles et al., 2021). This study attempted to use CVT as the theoretical framework to analyze the effects of achievement emotions on online learning outcomes in terms of motivation, engagement, performance, satisfaction, and achievement.

## Literature review, aims, and research questions

### Achievement emotions

#### Definitions of achievement emotions

Achievement emotions, also known as academic emotions, could be defined as emotions in relation to achievement activities and achievement outcomes, such as disappointment about unattainable learning goals (Pekrun, 2006). Achievement emotions could be deemed as either state emotions or trait emotions typically experienced in various academic environments (Pekrun et al., 2011). For instance, some learners might experience a high level of anxiety when taking exams, others when attending face-to-face classes. Achievement emotions are multifaceted processes that consist of cognitive, psychological, and motivational components (Fraschini and Tao, 2021). Unlike other emotions in educational contexts, achievement emotions involve specific object focuses and appraisal-driven psychological processes (Putwain et al., 2018).

#### Classifications of achievement emotions

Emotions could be categorized according to three dimensions, i.e., object focus, valence, and activation (Pekrun et al., 2011). In terms of object focus, activity emotions could be distinguished from outcome emotions. Regarding valence, positive emotions could be differentiated from negative emotions. As regards activation, activating emotions could be distinguished from deactivating emotions (Pekrun et al., 2002). According to the three dimensions, achievement emotions could thus be grouped into different categories, such as positive activating emotions and negative outcome emotions (Pekrun and Stephens, 2010; Pekrun et al., 2017). Table 1 displays an overview of a three-dimensional taxonomy of achievement emotions.

**Table 1:** A three-dimensional taxonomy of achievement emotions (Adapted from Pekrun and Stephens, 2010).

**Object focus**	**Positive emotions**		**Negative emotions**	
	**Activating**	**Deactivating**	**Activating**	**Deactivating**
Activity	Enjoyment	Relaxation	Anger Frustration	Boredom
Outcome/Prospective	Joy^*^ Hope	Relief^*^	Anxiety	Hopelessness
Outcome/Retrospective	Joy Pride Gratitude	Contentment Relief	Shame Anger	Sadness Disappointment

* Anticipatory joy/relief.

#### Previous reviews of achievement emotions

Recently, there have been some reviews of the effects of achievement emotions in education. In a comprehensive review, boredom experienced in traditional learning contexts was proven detrimental to achievement, motivation, and learning strategies (Tze et al., 2016). A systematic review revealed that enjoyment positively influenced learning outcomes in technology-enhanced contexts. It also mentioned that anger, frustration, and boredom merely had a slightly negative influence on learning outcomes (Loderer et al., 2020). Similarly, another literature review concluded that a positive correlation was found between enjoyment and performance, and negative correlations were found for both anger and boredom (Camacho-Morles et al., 2021). Meanwhile, Tan et al. (2021) reported that positive emotions were better than negative ones at enhancing learning effects.

Despite the increasing importance of achievement emotions in online learning (Yu et al., 2020), very few published studies have synthesized the effects of achievement emotions on online learning outcomes (see Table 2). Given that the effects of achievement emotions on online learning outcomes have been unclear, it is meaningful to systematically review publications reporting the effects of achievement emotions on online learning outcomes in terms of motivation, performance, engagement, satisfaction, and achievement.

**Table 2:** Previous reviews of achievement emotions.

**Source**	**Date range**	**Emotion(s)**	**Finding(s)**	**Context**
Tze et al. (2016)	1990–2014	Boredom	Achievement; Motivation; learning strategies	Traditional learning contexts
Loderer et al. (2020)	1965–2018	Enjoyment, curiosity/interest, anxiety, anger/frustration, confusion	Engagement; learning strategy; achievement	Technology–based learning contexts
Camacho-Morles et al. (2021)	1986–2019	Enjoyment, anger, frustration, and boredom,	performance	Not specified
Tan et al. (2021)	2012–2020	Not specified	Learning effects	Not specified
Innovation of this study	1986–2022	All emotions included in selected studies	Motivation; Performance; Satisfaction; Engagement; Achievement	Online learning contexts

### Motivation

Motivation, as a crucial variable in online learning, is the mental state that stimulates and maintains online learning behaviors (Yu, 2022). Motivation is generally divided into extrinsic and intrinsic motivation. The former refers to individuals' desire to do an activity in order to obtain some separable outcome, whereas intrinsic motivation refers to individuals' desire to do an activity in order to gain a sense of inherent satisfaction (Ryan and Deci, 2000). Motivation, in this study, was defined as the desire to participate in online learning activities.

Achievement emotions are important factors influencing learners' motivation (Pekrun, 2006). With the exponential growth in online learning, there has been a growing academic interest in the precise effects of achievement emotions on learning motivation (Lee J. et al., 2021). Evidence has suggested that different achievement emotions had diverse mechanisms for online learning motivation (Murphy and Rodriguez, 2008; Lee J. et al., 2021). However, not enough studies systematically reviewed the related literature. Considering the importance of motivation, it is meaningful to review and synthesize the effects of achievement emotions on online learning motivation.

### Performance

Performance is one of the most significant factors influencing online educational quality and success (Zhu et al., 2022). Performance could be generally described as individuals' learning attitudes and behaviors (Lu and Lin, 2016). Specifically, performance refers to how learners deal with their studies and how they accomplish learning tasks assigned by their instructors (Kayode, 2015). Performance also refers to the degree to which learners are continuing to learn in order to achieve learning goals (Eid and Al-Jabri, 2016). Performance in this study refers to how online learners cope with their learning materials and tasks.

Much of the available literature has focused on the influence of achievement emotions on performance in online learning contexts. However, inconsistent findings are still present with regard to the effects of achievement emotions. Online learners could improve their performance because they have experienced positive achievement emotions (Parker et al., 2021). Nevertheless, some students feeling positive emotions have performed poorly in online learning (Liu et al., 2021). To date, the effects of achievement emotions on online learning performance have still not been systematically reviewed.

### Engagement

Engagement could be deemed as sustaining efforts that learners make to achieve goals in academic learning (Jung and Lee, 2018). It is a multidimensional structure consisting of behavioral, cognitive, and emotional aspects. Behavioral engagement refers to an individual's participation in learning activities. Cognitive engagement is deemed as an individual's willingness to perform difficult tasks. Emotional engagement includes an individual's emotional reactions to learning (Fredricks et al., 2004). Engagement is regarded as a significant indicator of the quality of online education (Xu et al., 2020). Engagement in this study was defined as learners' involvement in online learning activities.

Numerous studies have examined whether achievement emotions could influence learners' engagement in online learning (e.g., Golding and Jackson, 2021). The existing findings on the precise effects of achievement emotion, however, have been contradictory. On the one hand, certain studies claimed that learners' engagement may be subject to learners' achievement emotions in online courses (D'Errico et al., 2016). More recently, certain studies have emerged that provided inconsistent findings. No significant relationship was found between learners' achievement emotions and their engagement in online learning (Wu et al., 2021a). The inconsistent findings were also supported by Wang et al. (2022). Given the inconsistent findings, it was necessary to conduct a systematic review.

### Satisfaction

Satisfaction could be operationally defined as the range of mental states where learners have the feeling of contentment with online learning experiences and online courses. Satisfaction also is an affective outcome influencing learners' intention to participate in online learning (Taghizadeh et al., 2021). Meanwhile, satisfaction is an important variable, as it is closely associated with stronger motivation, higher engagement, better performance, and even greater achievement in all learning contexts (Pike, 1991; Wu et al., 2021a). More importantly, learning satisfaction is an important indicator of learning outcomes (Alqurashi, 2018; Al-Fraihat et al., 2020). Therefore, it is of great significance to explore influencing factors in learning satisfaction (Yu, 2015).

Current literature has paid attention to the effects of achievement emotions on online learning satisfaction. The existing research reported that achievement emotions exerted a negative (Artino, 2009), positive (Golding and Jackson, 2021), and even non-significant (Wu et al., 2021a) influence on online learning satisfaction. In conclusion, these studies showed that the effects of achievement emotions on satisfaction were complex. One way to further understand the complex effects was to conduct a systematic review to summarize these existing findings.

### Achievement

Achievement could be deemed as learners' improvement in skills and comprehension of information (Ebel and Frisbie, 1986). Achievement is a criterion for the assessment of learners' competencies (Madigan and Curran, 2021). Achievement in this study could be deemed as online learners' academic success and learning gains. Achievement might be identified by two measures, i.e., perceived success and actual achievement. Perceived success refers to online learners' perceptions regarding their actual attainment while actual achievement refers to online learners' test scores.

Numerous studies have examined the effects of achievement emotions on online learning achievement (e.g., Pan et al., 2022). It was demonstrated that achievement emotions could in the least exert an influence on online learning achievement as other psychological factors. However, not enough evidence suggested which achievement emotions could lead to significantly higher online learning achievement, nor did evidence suggest whether the effects of achievement emotions on achievement might vary in different online courses.

### Aims and research questions

This study set out to systematically review and synthesize findings on the effects of achievement emotions on learners' online learning outcomes in terms of motivation, performance, engagement, satisfaction, and achievement. We proposed the five questions: (1) Could achievement emotions influence learners' online learning motivation? (2) Could achievement emotions influence learners' online learning performance? (3) Could achievement emotions influence learners' online learning engagement? (4) Could achievement emotions influence learners' online learning satisfaction? (5) Could achievement emotions influence learners' online learning achievement?

## Research methods

### Research design

This study is a systematic review of the effects of achievement emotions on learners' motivation, performance, engagement, satisfaction, and achievement in online learning contexts. This study took a four-step approach to identify and synthesize prior literature, providing a comprehensive understanding of the effects of achievement emotions on online learning outcomes. Firstly, this review searched Web of Science to collect relevant literature. Secondly, this review identified hot research themes and proposed research questions, using clustering and mapping techniques in the program VOSviewer. Thirdly, this review selected literature based on Preferred Reporting Items for Systematic Review and Meta-analysis (PRISMA) principles (Page et al., 2021). Finally, this review provided a comprehensive understanding of the effects of achievement emotions on online learning outcomes after synthesizing and analyzing the included literature.

### Research corpus

The researchers initially collected relevant studies by searching Web of Science on May 25th, 2022. Web of Science consists of various databases such as Science Citation Index Expanded (SCI-EXPANDED), Social Sciences Citation Index (SSCI), Arts and Humanities Citation Index (AandHCI), Conference Proceedings Citation Index-Science (CPCI-S), Conference Proceedings Citation Index-Social Science and Humanities (CPCI-SSH), Emerging Sources Citation Index (ESCI), Current Chemical Reactions (CCR-EXPANDED), and Index Chemicus (IC). It could, therefore, minimize selection bias and improve the representativeness of included studies (Yu et al., 2022).

The researchers initially obtained a total of 3143 results by keying in “distance learn^*^” OR “distance teach^*^” OR “e-learning” OR “remote learn^*^” OR “remote teach” OR “online learn^*^” OR “online teach^*^” OR “digital learn^*^” OR “digital teach^*^” OR “massive open online courses” OR “MOOC” (topic) and “anxiety” OR “shame” OR “anger” OR “enjoyment” OR “boredom” OR “hope” OR “pride” OR “joy” OR “frustration” OR “relief” OR “relaxation” OR “hopelessness” OR “contentment” OR “disappointment” OR “sadness” OR “gratitude” OR “positive affect^*^” OR “positive emotion^*^” OR “negative affect^*^” OR “negative emotion^*^” OR “achievement emotions” OR “academic emotions” OR “emotion^*^” (topic), ranging from the inception to May 25th, 2022.

**Figure 1:**
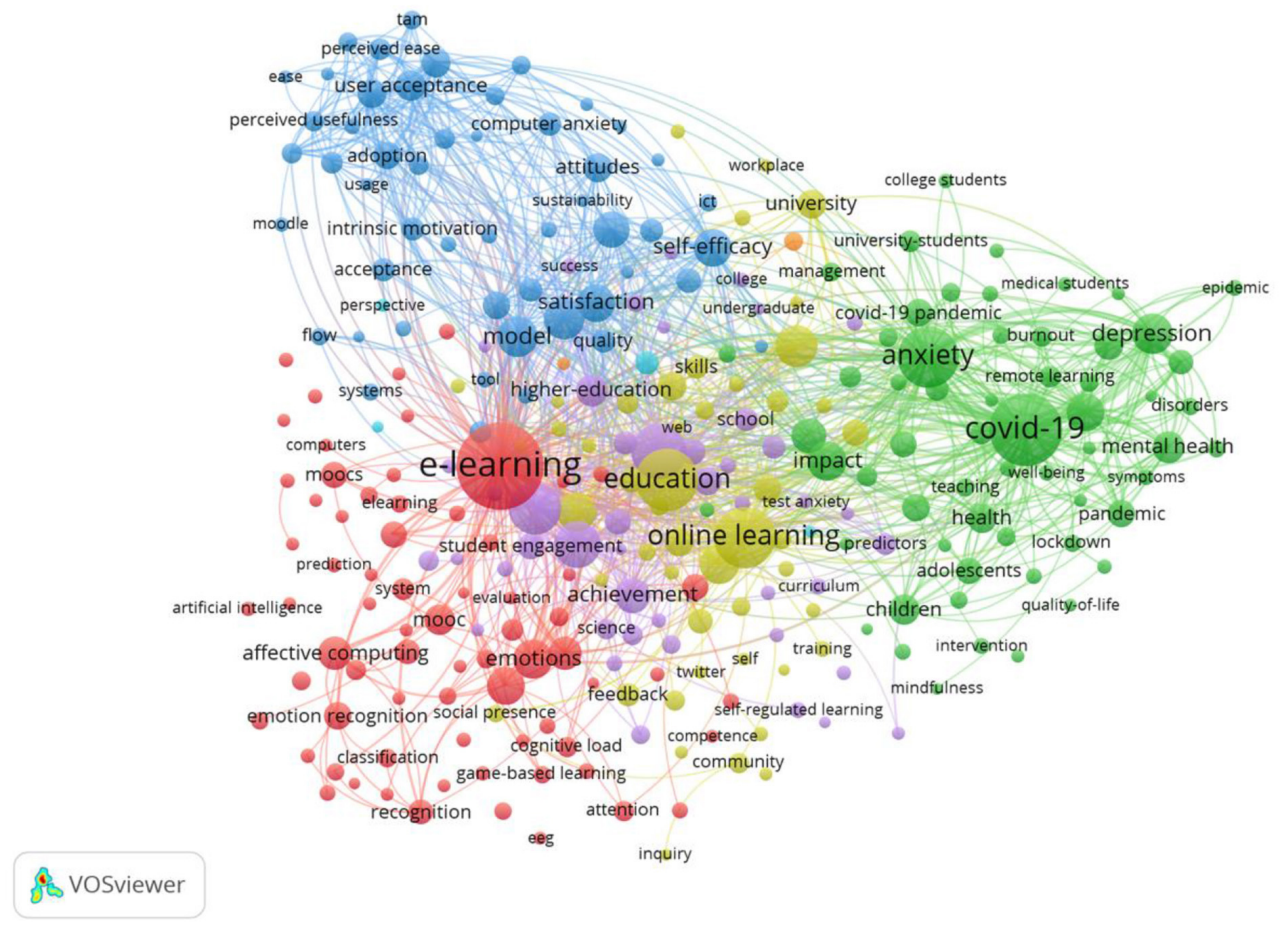
The bibliographic network.

To identify hot research themes in the collected literature, the researchers conducted a bibliographic network study using the program VOSviewer. Specifically, the researchers extracted the bibliographic data of the results (*N* = 3143) from Web of Science. Then, the researchers employed the program VOSviewer to interpret the bibliographic data, choosing co-occurrences as the analysis type, all keywords as the analysis unit, and full counting as the counting methods. The minimum number of occurrences of a keyword was set at 10. A total of 281 keywords met the threshold. Figure 1 provides an overview of the bibliographic network.

A total of 281 keywords were classified into 7 clusters. Cluster 1 included 71 items, e.g., academic emotion, achievement emotions, e-learning, massive open online courses, and virtual learning environments. Cluster 2 included 60 items, e.g., adolescents, adults, anxiety, burnout, children, and college students. Cluster 3 included 51 items, e.g., acceptance, adoption, attitudes, behavioral intention, and ease. Cluster 4 included 48 items, e.g., adaption, challenges, feedback, language, and knowledge. Cluster 5 included 45 items, e.g., satisfaction, performance, achievements, motivation, and student engagement. Cluster 6 included 4 items, e.g., accessibility, perspective, support, and teacher. Cluster 7 included 2 items, e.g., beliefs and instructional design.

The researchers identified the hot research themes based on a list of keywords with the top number of the co-occurrence links. The link strength of motivation (*N* = 960), performance (*N* = 873), satisfaction (*N* = 744), and engagement (*N* = 561) were highly ranked. The item achievement also had a strong link strength (*N* = 480). From the link strength, motivation, performance, engagement, satisfaction, and achievement are hot themes in this particular field.

### Inclusion and exclusion criteria

We followed the PRISMA principles to include and exclude the literature. Inclusion and exclusion criteria were formal categories. Publications were only included in the research if they (1) shed light on the effects of achievement emotions on motivation, engagement, performance, satisfaction, and achievement in online learning contexts, (2) provided adequate information and full texts for this research, (3) were written in English, (4) were well-designed journals or conference proceedings, (5) had reliable and valid findings, and (6) reached convincing conclusions. Publications were excluded if they (1) were duplicates, (2) were written in other languages, (3) did not include an acceptable abstract, (4) were reviewers, book chapters, books, book reviewers, data papers, editorial materials, meeting abstracts and unpublished articles, (5) focused on achievement emotions rather than their effects on motivation, engagement, performance, satisfaction, and achievement, and (6) could not provide enough statistical information.

### Study selection

**Figure 2:**
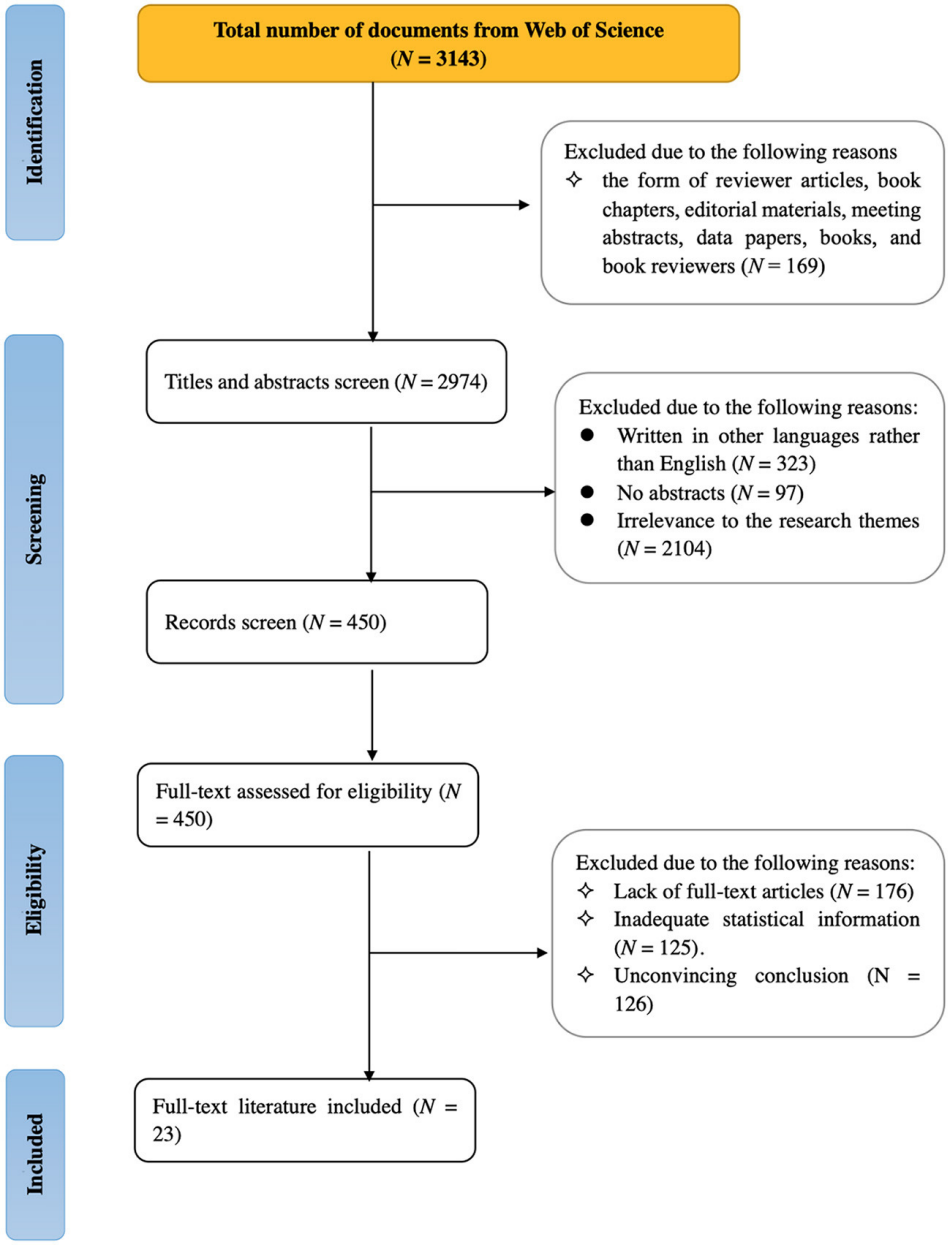
A flow diagram of the study selection based on PRISMA.

Two researchers screened the collected literature independently based on formal inclusion and exclusion criteria. There were four phases, as shown in Figure 2. The researchers initially obtained 3,143 publications from Web of Science. After checking the document types, the researchers removed reviewer articles (*N* = 88), book chapters (*N* = 50), editorial materials (*N* = 20), meeting abstracts (*N* = 5), data papers (*N* = 4), books (*N* = 1), and book reviewers (*N* = 1). After screening titles and abstracts, the researchers selected 450 publications for full-text review. Through evaluation of full-text publications for eligibility, the researchers finally included 23 publications for this systematic review. The Cohen's Kappa value was 0.93, which indicates high inter-rater reliability between the two researchers.

### Quality assessment

A quality assessment tool proposed by Kmet et al. (2004) was used to evaluate the quality of these selected publications. It includes two systems that could be applied to the evaluation of qualitative research and quantitative research respectively. Quantitative studies could be assessed by using 14 formal criteria, such as appropriate sample size and analytic methods. A total of 10 formal criteria were applied to the assessment of qualitative studies, such as sampling strategy and use of verification procedures. Categories of criteria were formal. Two researchers scored each publication independently (“yes” = 2, “partial” = 1, “no” = 0). The inter-rater agreement ranged from 60 % to 100%, suggesting acceptable quality.

### Data abstraction and synthesis

This review adopted the data abstraction and synthesis method designed by Bridges et al. (2020). Content analysis was applied to synthesize and extract the findings of included publications. There were three stages. In the first stage, two researchers carefully read the findings and results of included publications and extracted all data on samples, methods, analytic techniques, strengths and weaknesses of studies, and the effects of achievement emotions. Two researchers inductively coded data. High inter-coder reliability was found between two researchers (α = 0.90). In the second stage, researchers grouped codes together and put them into the following categories, i.e., engagement, satisfaction, motivation, satisfaction, and achievement. In the third stage, researchers provided a systematic understanding of the effects of achievement emotions on online learning motivation, performance engagement, satisfaction, and achievements.

### Descriptive information

Included publications were categorized based on their publication years (see Appendix). It could be seen that included publications were published from 2009 to 2022. The number of publications increased steadily before 2021. However, the number of publications has been rising more quickly since 2021. A possible explanation for this might be that the rapid transition to online education during the pandemic has led to an exponential growth of research on achievement emotions in online learning contexts.

The findings of descriptive statistics were shown in terms of samples, methods, and analytical techniques. Of the 23 publications reviewed, the majority of publications (*N* = 20) selected university students as samples, and two studies selected primary school students and high school students as samples respectively. Only one study selected students' forum posts as samples. Studies were conducted in the United States (*N* = 5), China (*N* = 4), Germany (*N* = 3), Italy (*N* = 3). There was one study in each of the following countries: Australia, Canada, Indonesia, Jamacia, the Netherlands, South Korea, and the United Kingdom (in alphabetical order). The sample sizes varied from 64 students to 400,000 forum posts. Correlation and regression analysis (*N* = 16) was found to be the most popular analytical technique to investigate the effects of achievement emotion on online learning outcomes. Other techniques used to a lesser extent were structural equation modeling (*N* = 8), factor analysis (*N* = 6), ANOVA (*N* = 5), and *t*-test (*N* = 3). Many scholars also employed qualitative approaches, such as latent profile analysis (*N* = 2), content analysis (*N* = 2), association rule mining techniques (*N* = 1), and sentiment analysis (*N* = 1).

## Results

This section concluded the effects of achievement emotions on online learning outcomes in terms of motivation, engagement, satisfaction, performance, and achievement. Table 3 shows the effects in detail. The symbol “+” shows that there is a positive effect. The symbol “–” suggests that there is a negative effect. The symbol “/” means that the research does not find the effects. The symbol “&” indicates that there are different findings. Blank space suggests that the relationships between factors were not discussed in the included studies.

**Table 3:** A summary of effects of different emotions.

	**Online learning outcomes**				
**Emotions**	**Motivation**	**Performance**	**Engagement**	**Satisfaction**	**Achievement**
**Enjoyment**	+	+	+ and /		+
**Pride**	+	–		+	+
**joy**	+			+	+
**Relaxation**		–			+
**Relief**		–		+	
**Hope**				+	+
**Gratitude**					
**Boredom**	–	+ and–	/	–	–
**Anxiety**	–	+		+ and–	–
**Frustration**	–	+	/	–	–
**Anger**					–
**Shame**					–
**Hopelessness**					–
**Sadness**					

The effects of achievement emotions on online learning outcomes are on the horizontal axes; discrete achievement emotions and indicators of online learning outcomes are on the vertical axes.

### RQ 1: Could achievement emotions influence online learning motivation?

#### The influence of achievement emotions on motivation

Generally, positive achievement emotions, such as enjoyment, pride, and joy, could increase online learning motivation. Promoting undergraduates' positive emotions of enjoyment and pride could be useful for the increase of their motivation in online medical mathematics courses (Kim and Hodges, 2012). Having a higher motivation, high school students reported a higher level of enjoyment and pride in online math courses (Kim et al., 2014).

In the contrast, negative emotions, such as anxiety, boredom, and frustration, have a detrimental impact on online learners' motivation. Being bored and frustrated, service academy undergraduates were not motivated to enroll in future online courses (Artino, 2009). Similarly, college students feeling bored reported that they lacked motivation in online courses (Parker et al., 2021). College students with negative emotions had a low level of learning motivation which may reduce the opportunities to gain an academic qualification (Heckel and Ringeisen, 2019).

### RQ2: Could achievement emotions influence online learning performance?

#### Complex effects

Evidence has suggested that in online learning contexts, the effects of achievement emotions on performance might be more complex. Evidence has suggested that positive emotions could be better than negative ones at enhancing online learning performance. Experiencing enjoyment in a virtual learning environment, international business students achieved better performance (Noteborn et al., 2012). Chinese college students also improved their learning performance in online learning environments when experiencing positive emotions of joy, hope, relaxation, and pride, similar to those in traditional classrooms (Zhu et al., 2022). Consistent with this finding, Parker et al. (2021) reported that high control-enjoyment students perceived themselves as successful and outperformed those with low control-boredom in a two-semester online course.

Nevertheless, different arguments still exist regarding the influence of achievement emotions on online learning performance. On the one hand, it was found that positive emotions were detrimental to online learners' performance. Positive deactivating emotions, such as relief and relaxation, reduced students' effort and use of proactive strategies, eventually leading to a greater risk of poor performance in MOOCs (Liu et al., 2021). Primary school students were likely to feel pride when they had false perceptions of their own performance in online learning. It might be detrimental to their performance in online learning (Raccanello et al., 2020).

On the other hand, negative activating emotions could be considered to be beneficial in terms of online learning performance. A Low level of anxiety could enhance students' performance and competence in online learning (Heckel and Ringeisen, 2019). Students, who felt bored with theoretical courses, performed better on practical assignments in virtual learning (Noteborn et al., 2012). Frustration could exert a positive influence on learners' performance in MOOCs because it could stimulate learners to make efforts to avoid failure (Liu et al., 2021). Many part-time distance learners perceived that anxiety could enhance their participation and performance in online collaborative learning because they were motivated by frustration to adopt problem-focused coping strategies (Hilliard et al., 2020).

#### Age and gender differences

Age could be the moderator between achievement emotions and online learning performance. Compared with younger learners, older learners had a lower level of pride but a better performance in digital tasks (Raccanello et al., 2020). However, gender seemed not to moderate the effects of achievement emotions on online learning performance. There was no significant difference between male students and female students in terms of online learning performance, despite that male students experienced a higher level of anxiety in online learning than female students (Mahande et al., 2021).

### RQ3: Could achievement emotions influence online learning engagement?

#### A much-debated topic

The influence of achievement emotions on online learning engagement might be a much-debated topic. Some studies identified the positive effects of positive emotions and negative effects of negative emotions. Positive achievement emotions could help Italian university students to engage in different online learning activities (D'Errico et al., 2016). Chinese college students with enjoyment tended to actively construct knowledge and avoid academic failure, thus having a higher level of engagement in online learning (Wang et al., 2022). Jamaican high school students, who felt frustrated and anxious, were less likely to be engaged in online learning (Golding and Jackson, 2021). However, others found that both positive and negative emotions could not influence online learners' engagement. The achievement emotions of enjoyment, boredom, and frustration did not exert an influence on students' engagement in MOOCs (Wu et al., 2021a). Similarly, Wang et al. (2022) found that frustration could not influence Chinese college students' engagement in online learning.

### RQ4: Could achievement emotions influence online learning satisfaction?

#### The influence of achievement emotions

The influence of achievement emotions on online learning satisfaction was examined. Several studies have established that positive emotions could play a crucial role in improving online learning satisfaction. Negative emotions negatively impacted online learning satisfaction. High school students, who have experienced joy, hope, pride, and relief, were more satisfied with online learning than those feeling frustrated, anxious, and bored (Golding and Jackson, 2021). Experiencing more enjoyment as well as less anxiety and boredom in online learning, Chinese pre-service teachers reported a higher level of satisfaction in online learning (Wu et al., 2021b). Also, college students, who felt happiness and pride in online learning, were much more satisfied with online learning (Zhu et al., 2022). By contrast, negative achievement emotions, such as sadness, frustration, and anxiety, have led undergraduate nursing students to feel dissatisfied with online courses (Santo et al., 2022). Similarly, undergraduates who reported boredom and frustration were not satisfied with online courses (Artino, 2009).

#### Contradictory evidence

Nevertheless, much literature has emerged that offered contradictory findings on the effects of negative emotions on satisfaction. University Students feeling a sense of pride were satisfied with online learning, whereas students who have experienced a low level of anxiety in online learning also reported a high level of learning satisfaction (Heckel and Ringeisen, 2019). Similarly, Korean undergraduates having a high level of negative emotions had a higher level of online learning satisfaction, compared to those reporting a medium level of negative emotions (Lee and Chei, 2020). Contrary to previous findings, Wu et al. (2021a) found no significant effects of achievement emotions on students' satisfaction with MOOCs.

### RQ5: Could achievement emotions influence achievement?

#### The effects of achievement emotions

Achievement emotions could play a crucial role in students' achievement in online learning. Positive achievement emotions were beneficial to learners' achievement, while negative emotions were detrimental to their achievement. Specifically, enjoyment was positively correlated to online learners' success in programs and technology use (Butz et al., 2015). On the other hand, evidence supported that negative achievement emotions were detrimental to online learning achievement. Anxiety and frustration may result in a low level of achievement for university students, particularly during the full-on digital semester (Stockinger et al., 2021). Korean undergraduates with a high level of boredom showed a lower level of perceived achievement in online learning than those with a high level of enjoyment (Lee and Chei, 2020).

#### Different online courses

Research on achievement emotions has been committed to exploring the roles of achievement emotions in various online courses, especially in language courses, business courses, and math courses. Achievement emotions could play a similar role in these online courses. In online language courses, Asia language learners with a higher level of enjoyment and pride had significantly higher achievement, compared to those with a higher level of anxiety (Fraschini and Tao, 2021). The increasing level of boredom was negatively associated with students' perceived achievement and GPA in online business courses (Butz et al., 2016). K-12 students, who got higher exam scores on math tests, were more likely to report a lower level of anxiety, anger, shame, and hopelessness but a higher level of enjoyment and pride in online mathematics courses (Kim et al., 2014).

#### Different findings

Gender differences in the effects of achievement emotions on achievement were investigated. Evidence showed that there was no significant difference between male students and female students with respect to online learning achievement, even though female students showed a higher level of hope than male students in online learning (Stephan et al., 2019).

## Discussion

The aim of this review was to explore whether achievement emotions could influence online learning outcomes in terms of motivation, performance, satisfaction, engagement, and achievement. A total of 23 publications were included in this review. As indicated previously, achievement emotions had different mechanisms for online learning motivation, performance, engagement, satisfaction, and achievement.

### The positive effects of positive achievement emotions

It was not surprising that positive achievement emotions, such as enjoyment, joy, pride, and relief, could exert a positive influence on online learners' motivation, engagement, satisfaction, performance, and achievement. In other words, positive achievement emotions are very important for improving online learning outcomes. These results support the findings of previous studies (e.g., Camacho-Morles et al., 2021). Experiencing positive achievement emotions, online learners were willing to participate in learning activities and interact with content, peers, and instructors. When they had more effective interactions in the learning activities, they were likely to actively construct knowledge, reflect on their own online learning experiences, and develop a sense of community and the ability to self-regulate (Wang et al., 2022). Consequently, they had a high level of learning motivation, engagement, satisfaction, performance, and achievement in online learning.

Nevertheless, it is worth noting that excessive positive emotions may lead to poor performance in online learning. Relief and relaxation might also reduce online learners' effort and use of proactive strategies, eventually leading to poor learning performance (Liu et al., 2021). Online learners, who were filled with pride, might overestimate their performance and then put less effort into learning activities, eventually having a greater risk of poor performance. The younger learners were more vulnerable to negative effects than the older learners (Raccanello et al., 2020). Therefore, instructors need to pay attention to online learners' positive emotions, especially younger learners.

### Difficulties in determining the effects of negative achievement emotions

A growing body of research has been committed to the effects of negative achievement emotions on online learners' motivation, engagement, performance, satisfaction, and achievement. However, it might be hard to determine the effects of negative achievement emotions. Some studies reported the negative influence of negative achievement emotions on motivation (Artino, 2009), engagement (Raccanello et al., 2020), performance (Parker et al., 2021), satisfaction (Golding and Jackson, 2021), and achievement (Stockinger et al., 2021), while some found that negative emotions, such as anxiety and frustration, could enhance learners' performance (Liu et al., 2021) and satisfaction (Heckel and Ringeisen, 2019). On the other hand, Wang et al. (2022) found no significant correlation between learning engagement and negative achievement emotions. These results broadly are in agreement with previous reviews (e.g., Tan et al., 2021).

Although scholars have long debated the impact of negative achievement emotions, it is important to notice that keeping negative achievement emotions under control might be conducive for online learners. A possible explanation might be that adequate levels of negative achievement emotions could stimulate learners to make efforts to practice and avoid failures, especially when learners were eager to have better performance and achievement in online learning (Loderer et al., 2020; Liu et al., 2021). Therefore, instructors need to be aware of online learners' negative emotions and provide online help to those learners.

## Conclusion

### Major findings

This study systematically reviewed research on the effects of achievement emotions on motivation, engagement, performance, satisfaction, and achievement in online learning contexts. The findings suggested that in online learning contexts, positive achievement emotions could be much more effective to improve learners' motivation, engagement, performance, satisfaction, and achievement, compared to negative achievement emotions. It is worth noting that negative activating emotions, such as anxiety and frustration, could be beneficial to online learners' performance and satisfaction. Keeping achievement emotions under control could have a beneficial effect on online learning motivation, performance, engagement, satisfaction, and achievement. Most importantly, multiple intervention strategies, such as teaching interventions, technological interventions, and treatment interventions, could be used to intervene in online learners' emotions and in turn benefit online learners academically.

### Educational implications

It would be more effective to adopt certain intervention strategies to encourage online learners to control and regulate achievement emotions, thereby mitigating their emotional barriers and improving online learning outcomes. Effective interventions include teaching interventions, technological interventions, and treatment interventions.

#### Teaching interventions

It has been essential to adopt teaching interventions to help learners control and regulate achievement emotions, which in turn promote learners' motivation, engagement, satisfaction, performance, and achievement in online learning contexts. Teaching intervention strategies could be provided in the following areas: (a) online learning environments and (b) course design.

One important approach to intervening in online learners' achievement emotions is to provide friendly and supportive online learning environments. To improve online learning outcomes, instructors need to build encouraging and supportive learning environments for online learners to express their emotions and acquire emotion-based coping strategies (Hilliard et al., 2020). Forming online peer support groups could provide learners with emotional support and a learner-centered atmosphere, which in turn positively influences learners' satisfaction (Lee M. et al., 2021). Teachers could create collaborative learning environments where online learners could develop a sense of community and experience enjoyment in online learning (Kohnke et al., 2021).

Well-designed online courses are essential to intervene in the potential issues associated with online learners' emotional experiences (Lee and Chei, 2020). To satisfy different emotional requirements, instructors could implement effective blended teaching strategies (Mahande et al., 2021). Providing detailed information on online course design could be beneficial for learners to perceive courses as useful and increase their positive emotions (Gopal et al., 2021). Integrating interesting examples and demanding activities into short clips of video lectures is an effective teaching strategy to induce online learners' positive emotions and increase their concentration (Lee J. et al., 2021). Learning topics and forums integrated with confusing information could foster online learners' curiosity, improving learners' performance in online learning (Liu et al., 2022). Videos with good instructor images could trigger learners' positive emotions and in turn help improve their satisfaction with online learning (Yuan et al., 2021).

#### Technological interventions

Using technological interventions could generally benefit online learners both emotionally and academically. Augmented reality (AR) and virtual reality (VR) applications could create fun and highly immersive learning experiences, which could relieve online learners' boredom and enhance their engagement (Cesari et al., 2021). User-friendly online learning systems could lead to positive emotions and engagement among online learners, which contributes to their achievement in online learning (Lee and Chei, 2020). Online educational games could be deemed as effective educational technologies not only to trigger positive emotions but also to improve learning outcomes (Tzafilkou and Economides, 2021). Emotion recognition technologies integrated with intelligent computing functions could automatically detect facial expressions and provide feedback on emotional states. It could be useful to help online learners regulate their emotional states so that they could be deeply immersed in online learning (D'Mello and Graesser, 2012; Kouahla et al., 2022).

#### Treatment interventions

Instructors could employ various treatment interventions to reduce online learners' negative emotions and increase their positive emotions. Emotion control treatment intervention could help online learners put more effort to learn how to control their own emotions and in turn improve their motivation in online learning (Kim et al., 2014). Attributional retraining (AR) may encourage online learners to use more controllable and usable causes for their failures, thus reducing their anxiety and enhancing academic achievement in online learning (Parker et al., 2021).

### Limitations

A number of limitations need to be noted regarding the present study. Firstly, this study could not collect all relevant publications because of the limitations of library sources. Moreover, the scope of the present study was limited. There were reciprocal relationships between achievement emotions and learning outcomes (Putwain et al., 2022). However, this study only focused on the influence of achievement emotions on online learning outcomes but did not discuss the influence of online learning outcomes on achievement emotions. Thirdly, the effects of achievement emotions on students' learning outcomes may vary across cultural-educational contexts (Liu et al., 2020). However, the moderating effects of the cultural-educational contexts were not analyzed in the present study, given that included publications did not investigate the moderating roles of cultural-educational factors. Finally, this study only collected publications written in English, considering that English is the most commonly used language in the world. Some high-quality studies written in other languages were excluded from this study.

### Future research directions

Cultural-educational contexts might influence the intensity of achievement emotions and modes of emotion display (Pekrun and Stephens, 2010; Hagenauer et al., 2016). Nevertheless, relatively little research has been committed to comparing whether online learners' emotional experiences would vary in cultural-educational contexts, and even less to exploring whether the effects of achievement emotions would vary across cultural-educational contexts. Further studies need to conduct more control experiments to investigate whether online learners in different cultural-educational contexts had different intensity of achievement emotions as well as emotion play modes. Besides, future studies will need to pay particular attention to whether the contextual factors could moderate the effects of achievement emotions on online learning outcomes. For example, researchers could explore whether online learners in individualistic countries could express positive emotions more frequently than those in collectivist countries, and how these differences may contribute to online learning outcomes.

Future studies could deepen our understanding by exploring the moderating roles of individual variables in the relationships between achievement emotions and online learning outcomes. Individual variables, such as personality traits, genders, and age, may play a crucial role in online learning outcomes. Individual variables may moderate the relationships between achievement emotions and online learning outcomes (Yu and Deng, 2022). However, little current literature has paid attention to the moderating roles of individual variables. It is an urgent need to further explore these moderators.

## Data availability statement

The original contributions presented in the study are included in the article/Supplementary material, further inquiries can be directed to the corresponding author/s.

## Author contributions

RW: conceptualized, designed, collected, analyzed data, wrote, proofed, and edited this article. ZY: conceptualized, designed, revised, edited, proofed, and polished this article. Both authors contributed to the article and approved the submitted version.

## Funding

This work was supported by MOOC of Beijing Language and Culture University (MOOC201902) (Important) Introduction to Linguistics; Introduction to Linguistics of online and offline mixed courses in Beijing Language and Culture University in 2020; Special fund of Beijing Co-construction Project-Research and reform of the Undergraduate Teaching Reform and Innovation Project of Beijing higher education in 2020-innovative multilingual + excellent talent training system (202010032003); The Fundamental Research Funds for the Central Universities, and the Research Funds of Beijing Language and Culture University (22YCX038).

## Conflict of interest

The authors declare that the research was conducted in the absence of any commercial or financial relationships that could be construed as a potential conflict of interest.

## Publisher's note

All claims expressed in this article are solely those of the authors and do not necessarily represent those of their affiliated organizations, or those of the publisher, the editors and the reviewers. Any product that may be evaluated in this article, or claim that may be made by its manufacturer, is not guaranteed or endorsed by the publisher.
