# Supplementary material for: Exploring the effects of achievement emotions on online learning outcomes: A systematic review
Source: Front Psychol. 2022 Sep 9;13:977931. doi: 10.3389/fpsyg.2022.977931 (PMC9505900; doi:10.3389/fpsyg.2022.977931)
Supplement: Supplementary file 1 [file Table_1.docx]

**Appendix**

*Supplementary Table 1 A Summary of Included Publications*

| Author | Samples | Methods | Analytical techniques | Topics | Findings | Strengths | Weaknesses |
| --- | --- | --- | --- | --- | --- | --- | --- |
| Artino (2009) | 481 undergraduates from a US service academy | Questionnaires | Confirmatory factor analysis;  correlation and regression analysis | Satisfaction; Motivation | Boredom and frustration negatively impacted online learning satisfaction and motivation. | The study provided new insight into the relationships between negative emotions and motivation using a social-cognitive model. | The roles of positive emotions were not discussed. |
| Kim and Hodges (2012) | 91 students from a US university | Questionnaires | MANOVA | Motivation | Positive emotions could positively affect motivation. | The study used a comparison control group design to explore the effects of achievement emotions. | It was possible that these results might be bias due to a small number of participants. |
| Noteborn et al. (2012) | 155 international business students in the Netherlands | Questionnaire; Final exams | Regression analysis | Performance | Enjoyment had a positive effect on exam performance, whereas boredom had a positive influence on team assignments. | The study shed light on the effects of achievement emotions on academic success in a virtual world. | Learners were obliged to complete learning activities, which may impact students’ perceptions of emotions and therefore the possible bias in their responses. |
| Kim et al. (2014) | 72 students from a virtual high school in the USA | Questionnaires | Correlation and regression analysis | Achievement;  Motivation | Boredom, enjoyment, and anger could predict online learning achievement. | Researchers paid attention to the achievement emotions of high-school students. | The study failed to investigate the changes in achievement emotions over a long period. |
| Butz et al. (2015) | 218 graduate business students from the USA | Questionnaires | Confirmatory factor analysis; t-tests; correlation and regression analysis | Achievement | Emotions were closely related to program achievement. | Researchers explored the relationships between emotions and achievement in the synchronous hybrid program. | The study used single-item measure to evaluate learners’ emotions, which may reduce reliability. |
| Butz et al., (2016) | 118 university students from the U.S. | Questionnaires | Confirmatory factor analysis; t-tests; correlation and regression analysis; ANOVA | Achievement | Enjoyment positively influenced online learners’ perceived success and GPA, while anxiety and boredom negatively influenced their perceived success and GPA. | Researchers investigated the roles of students’ emotions in MBA and MPA programs longitudinally. | A limited number of participants led to the inconsistent results observed in mediation analyses. |
| D’Errico et al. (2016) | 78 Italian university students | Questionnaires | Correlation and regression analysis; ANOVA; t-test; | Engagement | Positive achievement emotions were associated with the increase in engagement in different online learning contexts. | The pilot study paid particular attention to various learning activities that might stimulate different types of emotions. | The pilot study was limited by the small sample and methodological rigor. |
| Heckel and Ringeisen, (2019) | 220 students from a German university | Questionnaires | Structural equation modeling | Satisfaction; performance | Pride positively influenced satisfaction and performance, whereas anxiety negatively influenced satisfaction in online learning settings. | The study formed an integrative approach to the investigation of emotions through combining CVT and SCT. | The responses relating to performance were subjective and thus led to a retrospective recall bias. |
| Stephan et al. (2019) | 182 German university students | Questionnaires | Confirmatory factor analysis; t-tests; correlation analysis; ANOVA | Achievement | Gender did not mediate the relationship between achievement emotions and achievement. | The study explored the moderating role of gender in the effects of achievement emotions on achievement. | Researchers did not exam whether or not there was a significant difference between females and males before the experiment. |
| Hilliard et al. (2020) | 76 students from The Open University UK | Questionnaires | correlation analysis; thematic analysis | Performance | Anxiety had different effects on students’ performance in online collaborative learning. | The combination of correlation analysis and thematic analysis was used to explore the effects of achievement emotions. | The options depicting positive effect of anxiety in the online surveys were deleted. |
| Lee and Chei (2020) | 777 South Korean undergraduates | Questionnaires; emotional profiles | Correlation and regression analysis;  latent profile analysis | Satisfaction; Achievement | Positive emotional experiences and few negative emotional experiences were associated with high perceived achievement and satisfaction. | The study provided greater insight into a reliable analytical method for students’ achievement emotions in online learning contexts. | The effects of individual variables and contextual variables were not explored in the study. |
| Raccanello et al. (2020) | 64 primary school students from Italy | Questionnaires; tests | Structural equation modeling ANOVA | Performance | Relaxation positively influenced performance, whereas pride and sadness negatively influenced performance in web-based learning. | The study compared students in different ages. | It may introduce a possible bias because the study did not exam whether there was the difference between groups regarding prior knowledge. |
| Fraschini and Tao (2021) | 117 students in an Australian university | Questionnaires | Correlation and regression analysis | Achievement | Both students’ positive emotions and negative emotions impacted their perceived learning gains and test scores. | Researchers measured the changes in students’ achievement emotions during four weeks. | Only a small number of participants completed all four surveys. It may suffer from a high degree of sampling bias. |
| Golding & Jackson (2021) | 486 high school students in Jamacia | Questionnaires; interviews | Exploratory factor analysis; regression analysis; content analysis | Satisfaction;  engagement | Students feeling positive emotions were more engaged in and satisfied with online learning, compared to those feeling negative emotions. | A combined qualitative and quantitative methodological approach was used to investigate the effect of emotions. | The study did not recruit representative samples. |
| Mahande et al. (2021) | 270 students from an Indonesian university | Questionnaires | Structural equation modeling | Performance | Anxiety positively impacted students’ performance in web-based learning. | This study considered individual differences in achievement emotions. | The study was limited by insufficient sample size. |
| Liu et al. (2021) | 400,000 Forum posts | 13 MOOCs | Sentiment analysis | Performance | Achievement emotions had different effects on students’ performance in MOOCs. | The study offered a perspective of achievement emotions using the data collected from forum posts | The study manually selected students’ forum posts from MOOCs, which may cause possible bias in the selection of posts. |
| Parker et al. (2021) | 327 students from a Canadian university | Questionnaires; tests; profiles | Latent Profile Analysis | Motivation;  Performance | High control-enjoyment students had better performance, compared to low control boredom students. | Researchers used person-center approaches examining the effects of achievement emotions over the two-semester curses. | The results of the study were not generalizable. It suggested that the findings needed to be confirmed in various online learning settings. |
| Stockinger et al. (2021) | 89 students in a South-German university | Questionnaires; tests | Structural equation modeling | Achievement | Achievement emotions were significantly associated with test scores and perceived learning outcomes. | The study shed light on the moderating roles of the situational factors in the effects of achievement emotions in the COVID-19 pandemic. | Random error was found in this research due to nonrandom sampling. |
| Wu et al. (2021a) | 283 pre-service teachers in China | Questionnaires;  Forums | Structural equation modeling; correlation analysis | Engagement; Satisfaction | Achievement emotions could not predict online learning engagement and satisfaction. | The study combined the CVT and UTAUT model to discuss the effects of achievement emotions. | Researchers did not discuss achievement emotions thoroughly. |
| Wu et al. (2021b) | 363 pre-service teachers in China | Questionnaires | Structural equation modeling; correlation analysis | Satisfaction  Engagement | Enjoyment improved online learning satisfaction. | The study explored the moderating role of gender in the effects of emotions. | The study was over dependent on self-report methodology. |
| Santo et al. (2022) | 353 students in an Italian university | Questionnaires | Structural equation modeling | Satisfaction | Emotions impacted learners’ satisfaction with online learning. | Researchers made contribution to research of emotions in e-learning nurse education. | The study did not evaluate the dynamic of affective states through longitudinal studies. |
| Wang et al. (2022) | 515 students from a university in central China | Questionnaires | Structural equation modeling; correlation analysis | Engagement | Enjoyment and boredom were associated with online learning engagement. | The study explored the complex relationships between interaction, achievement emotions, and engagement. | Researchers failed to detailed information on the effects of achievement emotions on different types of engagement. |
| Zhu et al. (2022) | 1088 Chinese college students | Questionnaires | Confirmatory factor analysis; correlation analysis | Performance;  satisfaction | Positive achievement emotions exerted a positive influence on students’ online learning achievement and satisfaction. | Researchers investigated the mediating roles of learning methods in the effects of achievement emotions. | The study only showed the casual relationships between factors. The study did not explore the relationships deeply. |
